# Two birds with one stone: pre-TAVI coronary CT angiography combined with FFR helps screen for coronary stenosis

**DOI:** 10.1186/s12880-025-01704-2

**Published:** 2025-05-26

**Authors:** Ruihui Wang, Dihao Pan, Xinlei Sun, Genren Yang, Jianjun Yao, Xiaoyong Shen, Wenbo Xiao

**Affiliations:** 1 Department of Radiology, The First Affiliated Hospital, Zhejiang University School of Medicine, Hangzhou, 310003 China; 2 Department of Cardiovascular Surgery, The First Affiliated Hospital, Zhejiang University School of Medicine, Hangzhou, China

**Keywords:** Coronary computed tomography angiography, Computed tomography fractional flow reserve, Coronary artery stenosis, Transcatheter aortic valve implantation, Diagnostic accuracy

## Abstract

**Objectives:**

Since coronary artery disease (CAD) is a common comorbidity in patients with aortic valve stenosis, invasive coronary angiography (ICA) can be avoided if significant CAD can be screened with the non-invasive coronary CT angiography (cCTA). This study aims to evaluate the ability of machine learning-based CT coronary fractional flow reserve (CT-FFR) derived from cCTA to aid in the diagnosis of comorbid CAD in patients undergoing transcatheter aortic valve implantation (TAVI).

**Methods:**

A total of 100 patients who underwent both cCTA and ICA assessments prior to TAVI procedure between January 2021 and July 2023 were included. Coronary stenosis was assessed using both cCTA data and machine learning-generated CT-FFR image information for patients/major coronary vessels. Coronary lesions with CT-FFR ≤ 0.80 were defined as hemodynamically significant, with ICA serving as the diagnostic gold standard.

**Results:**

A total of 400 major coronary vessels were identified in 100 eligible patients who underwent TAVI. CT-FFR was 86.4% sensitive and 66.1% specific to diagnose CAD, with a positive predictive value (PPV) of 66.7% and a negative predictive value (NPV) of 86.0%. The diagnostic accuracy (Acc) was 75.0%, with a false positive rate (FPR) of 33.9%. At the vessel level, CT-FFR showed a sensitivity of 77.6% and a specificity of 76.9%. The PPV was 44.0% and the NPV was 93.6%. The Acc was 77.0% and the FPR was 23.1%. For all patient/vessel units, CT-FFR outperformed cCTA.

**Conclusion:**

Machine learning-based CT-FFR can effectively detect coronary hemodynamic abnormalities. Combined with preoperative cCTA in TAVI patients, it is an effective tool to rule out significant CAD, reducing unnecessary coronary angiography in this high-risk population.

**Clinical trial number:**

Not applicable.

**Supplementary information:**

The online version contains supplementary material available at 10.1186/s12880-025-01704-2.

## Introduction

With the aging population, the prevalence of structural heart disease follows an increasing trend [1, 2, 3]. Among these, aortic valve stenosis (AS) poses a particularly serious clinical threat, demonstrating a very poor prognosis once the patient develops clinical symptoms, with a 2-year survival rate of 50% and a 5-year survival rate of only 20% [4]. Transcatheter aortic valve implantation (TAVI) is a catheter-based technique for treating AS that offers the advantages of minimal invasiveness and quicker recovery, making it suitable for patients unwilling or unable to undergo open-heart surgery [5].

Preoperative coronary CT angiography (cCTA) is required to assess the valve status of patients undergoing TAVI. Clinically, patients with severe AS often have comorbidities such as coronary artery disease (CAD), and some of them have a high plaque burden [4, 6], which may lead to a false-positive result on cCTA. Patients with more than 50% coronary stenosis on cCTA require further invasive coronary angiography (ICA) to clarify the coronary status. In clinical practice, ICA remains the gold standard for the diagnosis of coronary artery disease, but its invasive nature carries potential operative risks, including puncture site hematoma, arterial occlusion, perforation, arrhythmia [3, 7, 8], and acute kidney injury due to excess iodinated contrast agent [9], which limits its use in elderly and frail patients.

Although complex CAD is associated with worse 5-year outcomes after TAVI [10], non-selective angio-guided percutaneous coronary intervention remains controversial because it does not always significantly improve prognosis and may be risky [11, 12]. Therefore, improving the diagnostic accuracy of preoperative cCTA in patients planned for TAVI could aid clinical decision making and avoid unnecessary ICA. cCTA has been reported to have an accuracy and specificity of more than 80%, but its accuracy is significantly reduced in the presence of high coronary plaque burden [13]. To improve the diagnostic ability of cCTA, CT fractional flow reserve (CT-FFR) has been developed to non-invasively quantify the hemodynamic changes at the corresponding stenotic lesion site in patients with CAD. CT-FFR has also been reported to have higher accuracy in the diagnosis CAD [4, 13, 14].

The aim of this study was to evaluate the diagnostic value of CT-FFR based on preoperative cCTA for the diagnosis of CAD in patients scheduled for TAVI.

## Methods

### Study population

**Fig. 1:**
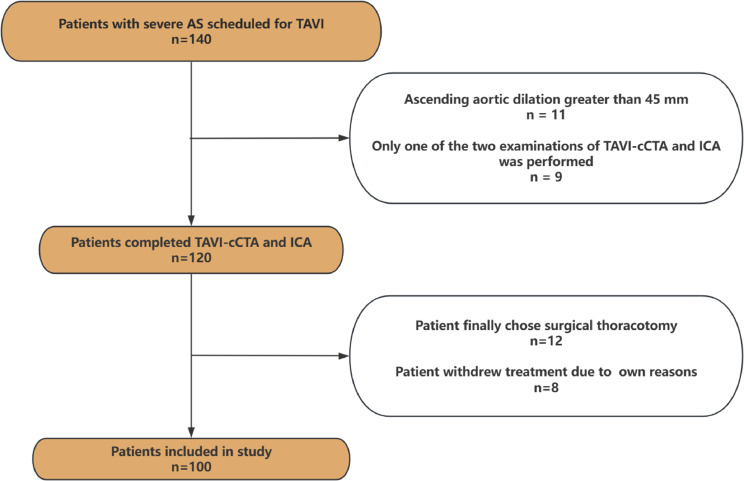
Flow chart for patient inclusion in the study

This is a retrospective clinical study. Patients (n = 140) planned to undergo TAVI between January 2020 and July 2023 at the First Affiliated Hospital, Zhejiang University School of Medicine were screened. Inclusion criteria were: (1) over 18 years of age; (2) diagnosed with severe aortic valve stenosis or regurgitation; (3) planned to undergo TAVI. Exclusion criteria for the study were: (1) aortic root anatomy unsuitable for TAVI; (2) only one of the two examinations, TAVI-cCTA and ICA, was performed; (3) severe comorbidities such as CAD requiring concomitant revascularization, severe mitral regurgitation, etc.; and (4) the patient ultimately opted for open-heart surgery. A total of 100 patients were included in the final analysis (see Fig. 1). The study protocol was approved by Clinical Research Ethics Committee of our center (IIT No. 2024–0305A) in accordance with the Declaration of Helsinki.

### CCTA imaging

The cCTA examination was performed on three spiral CT, namely Philips iCT, GE Revolution, and Siemens Force, using Onepac iodine as contrast agent at a blood flow of 5 ml/s, a blood volume of 50–55 ml, and 20–25 ml of saline pushed hydrostatically at the same flow rate. The scanning conditions were 100kV Tube voltage and automatic milliampere second tube current. The patients were instructed to hold their breath at the end of inspiration, and axial images were acquired from the apex of the heart to the base of the mediastinum. All the patients examined were gated using retrospective electrocardiography and reconstructed at R–R intervals every 10% to obtain TAVI-cCTA images.

The TAVI-cCTA image data were sent to a dedicated post-processing workstation (Shukun workstation), where two radiologists and a senior cardiology specialist worked together to visually analyze the morphologies of individual coronary vessels with the help of axial data and multiplanar reconstruction. The lesion characteristics of the discordant cases were discussed to reach conclusions by consensus. According to the CAD-RADS classification [14, 15], Coronary arteries with a diameter of 1.5 mm were analysed for the presence of CAD and the severity of stenosis: 0 (no evidence of CAD), 1 (minimal stenosis < 25%), 2 (mild stenosis 25–49%), 3 (moderate stenosis 50–69%), 4A (severe stenosis 70–99% in one or both vessels), 4B (≥50% stenosis in the left main trunk or ≥ 70% in three vessels stenosis), and 5 (complete occlusion).

Percentage stenosis was assessed for each lesion using both diameter-based and area-based methods, and the higher value was used to assess the overall CAD-RADS grade. Data on the degree of stenosis were obtained from cCTA in all patients; the presence of CAD was defined as moderate or greater stenosis (≥50% stenosis) in any of the four major coronary arteries (left main [LM], left anterior descending [LAD], left circumflex [LCX], and right coronary artery [RCA]).

### Machine learning-based CT-FFR computation

**Fig. 2:**
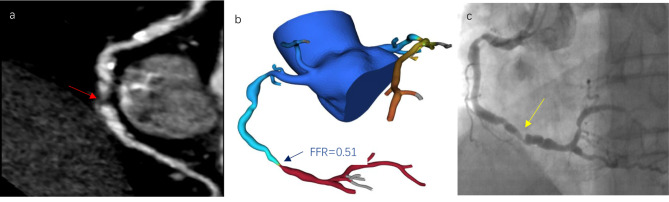
An illustrative case (cCTA, CT-FFR, ICA). Example of coronary stenosis on cCTA and ICA with corresponding CT-FFR using machine learning. cCTA in a patient prior to TAVR. (**a**) cCTA; (**b**) CT-FFR; (**c**) ICA. *cCTA*, coronary computed tomography angiography; *CT-FFR*, computed tomography-fractional flow reserve; *ICA*, invasive coronary angiography

Using ShuKun-FFR coronary analysis software, the fluid dynamics of the main branch vessels of the coronary artery were analyzed by a deep learning approach (see Fig. 2). The computational principle can be summarized in the following two core steps.

1. Coronary artery reconstruction:
  i. Correcting motion artifacts in CT scans using deep learning-based generative adversarial networks to improve image quality and diagnostic accuracy.
  ii. Coronary reconstruction of CT images using an improved U-net model, which is widely used for medical image segmentation tasks.
  iii. A 3D + 2D convolutional neural network model is applied for plaque detection and segmentation on processed CT images to obtain accurate geometric features of coronary artery lumen.
2. Computing Functional FFR: FFR is computed using a reduced-order model with machine learning prediction correction.
  i. The coronary arteries were divided into stenotic and non-stenotic regions, and the pressure differences between the regions were calculated using regional lumen geometry.
  ii. The computed pressures were further processed using a neural network to minimize the pressure difference between the reduced-order model results and the 3D computational fluid dynamics (CFD) or invasive measurements.

FFR values were collected as evaluation criteria for assessing the degree of coronary stenosis in patients/major vessels (threshold = 0.80).

### ICA

ICAs were performed by experienced cardiologists via radial or femoral artery access (including selective coronary cannulation), while quantitative coronary angiography (QCA) was used to determine the degree of coronary artery stenosis, performed using the QCA analysis module integrated into the Philips Azurion 7M12 digital subtraction angiography (DSA) system. Stenosis severity was categorized as: mild (<50%), moderate (50–69%), and severe (≥70%) [15]. Information from patients’ coronary angiograms was collected, and ≥ 50% stenosis in one of the major coronary arteries was diagnosed as CAD

### Statistical analysis

Statistical analyses were performed with SPSS software (version 27.0). The diagnostic performance of cCTA and CT-FFR was evaluated against ICA as the reference standard at both patient and vessel level. The corresponding true negative (TN), true positive (TP), false negative (FN) and false positive (FP) results were counted, and sensitivity (Sen), specificity (Spe), positive predictive value (PPV), negative predictive value (NPV), accuracy (Acc) and false positive rate (FPR) were calculated based on the following definitions.

- True positive (TP): ICA positive, cCTA/CT-FFR positive
- True negative (TN): ICA negative, cCTA/CT-FFR negative
- False positive (FP): ICA negative, cCTA/CT-FFR positive
- False negative (FN): ICA positive, cCTA/CT-FFR negative

$$Sen = \frac{{TP}}{{TP + FN}}\,$$

$$Spe = \frac{{TN}}{{TN + FP}}$$

$$PPV = \frac{{TP}}{{TP + FP}}$$

$$NPV = \frac{{TN}}{{TN + FN}}$$

$$Acc = \frac{{TP + TN}}{{Total\,patient}}$$

$$FPR = \frac{{FP}}{{TN + FP}}$$

## Results

### Patient clinical characteristics

The study ultimately included 100 patients who were proposed to undergo TAVI, of whom 46 (46.0%) had comorbid CAD. Analysis of the clinical characteristics of the patients revealed a mean age of 71.5 years old and a predominance of males (65.0%), as shown in Table 1. In addition, half of the patients were comorbidly hypertensive, 11% had comorbid diabetes mellitus, and some of the patients were smokers (21.0%) or alcohol drinkers (16.0%). The mean level of LDL-C was 2.1 ± 0.8 mmol/L. Patients with comorbid CAD tended to be slightly older than those without CAD (73.3 ± 8.5 vs. 69.9 ± 7.8, P < 0.05). No significant differences were found in the remaining characteristics.

**Table 1:** Baseline characteristics

	All patients (n = 100)	Negative CAD (n = 54)	Positive CAD (n = 46)	P value
Age (years)	71.5 ± 8.2	69.9 ± 7.8	73.3 ± 8.5	0.036*
Gender				0.222
Female	35 (35.0%)	16 (29.6%)	19 (41.3%)	
Male	65 (65.0%)	38 (70.4%)	27 (58.7%)	
Hypertension	50 (50.0%)	27 (50.0%)	23 (50.0%)	1.000
Diabetes	11 (11.0%)	3 (5.6%)	8 (17.4%)	0.059
Smoking history	21 (21.0%)	13 (24.1%)	8 (17.4%)	0.414
Alcohol history	16 (16.0%)	11 (20.4%)	5 (10.9%)	0.196
LDL-C(mmol/L)	2.1 ± 0.8	2.1 ± 0.7	2.1 ± 0.9	0.864

*CAD*, coronary artery disease; *LDL-C*, low-density lipoprotein cholesterol

### CT-FFR vs cCTA by patient

Based on the ICA results, the diagnostic efficacy of cCTA and CT-FFR were evaluated separately on a patient-by-patient basis (shown in Table 2). CT-FFR diagnosed CAD with Sen and Spe of 86.4% and 66.1%, respectively, PPV and NPV of 66.7% and 86.09%, respectively, Acc of 75.0%, and FPR of 33.9%. Results indicated that CT-FFR was superior to cCTA in majority of indicators but were slightly inferior in Spe and FPR.

**Table 2:** Diagnostic efficacy of cCTA and CT-FFR

	n	TP	TN	FP	FN	Sen	Spe	PPV	NPV	Acc	FPR
Patient cCTA	100	24	39	15	22	52.2%	72.2%	61.5%	63.9%	63.0%	27.8%
Patient CT-FFR	100	38	37	19	6	86.4%	66.1%	66.7%	86.0%	75.0%	33.9%
Vessels cCTA	400	39	241	95	25	60.9%	71.7%	29.1%	90.6%	70.0%	28.3%
Vessels CT-FFR	400	59	249	75	17	77.6%	76.9%	44.0%	93.6%	77.0%	23.1%
LM cCTA	100	1	84	14	1	50.0%	85.7%	6.7%	98.8%	85.0%	14.3%
LM CT-FFR	100	5	75	19	1	83.3%	79.8%	20.8%	98.7%	80.0%	20.2%
LAD cCTA	100	23	30	40	17	57.5%	42.9%	36.5%	63.8%	53.0%	57.1%
LAD CT-FFR	100	19	40	30	11	63.3%	57.1%	38.8%	78.4%	59.0%	42.9%
LCX cCTA	100	10	64	13	3	76.9%	83.1%	43.5%	95.5%	74.0%	16.9%
LCX CT-FFR	100	14	69	14	3	82.4%	83.1%	50.0%	95.8%	83.0%	16.9%
RCA cCTA	100	5	63	28	4	55.6%	69.2%	15.2%	94.0%	68.0%	30.8%
RCA CT-FFR	100	21	60	15	4	84.0%	80.0%	58.3%	93.8%	81.0%	20.0%

*cCTA*, coronary computed tomography angiography; *CT-FFR*, computed tomography-fractional flow reserve; *LAD*, left anterior descending; *LCX*, left circumflex; *LM,* left main coronary artery; *RCA,* right coronary artery; *TN*, true negative; *TP*, true positive; *FN*, false negative; *FP*, false positive

### CT-FFR vs cCTA by vessel

The diagnostic efficacy of CT-FFR was further evaluated in terms of the major coronary vessels (400 in total) (Table 2). CT-FFR was superior to cCTA in terms of overall evaluation indexes, with the Sen and Spe of 77.6% and 76.9%, the PPV and NPV of 44.0% and 93.6%, the Acc of 77.0%, and the FPR of 23.1%, respectively. The four branch vessels were analyzed separately, and it was found that both CT-FFR and cCTA performed poorly in the diagnosis of anterior descending branches, with FPR as high as 42.9% and 57.1%. Additional diagnostic performance is shown in Supplementary Table 1.

### Disscussion

In this study, we evaluated the diagnostic efficacy of preoperative CTA-derived CT-FFR for the detection of hemodynamically significant CAD in patients undergoing TAVI and provided recommendations for preoperative screening. Our results showed that CT-FFR outperformed CTA overall and had a high diagnostic value for exclusion. However, due to some false-positives, further ICA is required to confirm certain diagnoses, especially in those patients with severe coronary calcification.

CAD is a common complication in patients with severe AS. Considering the negative impact of proximal coronary stenosis on TAVI surgery and the coronary access problem associated with prosthetic valve implantation [1, 2, 4, 16], improving the systematic preoperative screening of patients is of clinical importance. As a routine test before TAVI, cCTA has a high negative predictive value for the diagnosis of CAD. cCTA-negative cases can be assessed as having normal coronary arteries without the need for ICA. However, in cCTA-positive patients with a high burden of calcified plaque in the coronary arteries, the false-positive rate of cCTA is significantly increased and the accuracy is greatly reduced.

Guidelines recommend ICA as the gold standard for the diagnosis of coronary artery disease [7, 8, 17, 18, 19], and it is now routinely performed in some centers. However, ICA is an invasive procedure with complications such as puncture site hematoma, arterial dissection, perforation, arrhythmia and other operative risks [20, 21]. In AS patients with comorbid chronic kidney disease, the risk of iodinated contrast overdose cannot be ignored [15, 17]. In addition, ICA is associated with additional radiation exposure, with a mean dose of 6–10 mSv (comparable to chest CT). Therefore, indiscriminate ICA is not recommended when cCTA positivity is suspected. This study evaluated the ability of cCTA and CT-FFR to detect CAD in patients with severe AS and showed that cCTA-based CT-FFR was superior to cCTA in terms of accuracy in detecting CAD and could be used as a tool for preoperative assessment of the coronary artery, avoiding unnecessary ICA.

The diagnostic value of CT-FFR in CAD has been explored in several studies. Budoff’s study [22] showed that CT-FFR was effective in predicting myocardial infarction. Decker found that machine learning based CT-FFR combined with a positive CTA result was effective in identifying CAD and may appropriately avoid unnecessary ICA. Serfaty [21] further investigated the clinical value of CT-FFR in the liver transplant population. In this study, we compared the ability of cCTA-based FFR with cCTA alone to identify patients with comorbid CAD based on routine preoperative cCTA in patients with severe AS undergoing TAVI. Comparatively, whereas previous studies used only one spiral CT device to collect data, the present study used multi-row spiral CTs from three different manufacturers, thereby improving the generalizability of the findings. In addition, unlike the non-standardized CT-FFR algorithm of Decker, this study used the commercially available ShuKun-FFR coronary analysis software to analyze the main branches of the coronary arteries to obtain CT-FFR values, with a stable platform and standardized study procedure, which is conducive to generalization.

However, this study has several limitations. First, routine invasive FFR measurement of all coronary branches was not feasible due to cost and procedural complexity, potentially introducing selection bias in the assessment of significant lesions. Future studies should implement a systematic FFR assessment protocol in all major coronary branches to improve methodological rigor and minimize bias. Second, current CT-FFR technologies include three approaches: 3D simulation-based modelling, simplified flow calculation, and deep learning-based analysis. This study focused exclusively on the deep learning-based approach, highlighting the need for future comparative studies to identify optimal methodologies. Finally, the interpretation of CT-FFR cut-off values in patients with severe AS is challenging due to significant changes in coronary hemodynamics before and after TAVI. Future protocols should consider these physiological transitions by broadening diagnostic thresholds and incorporating post-TAVI flow prediction into clinical decision making.

Furthermore, recent studies provide important insights to help refine the coronary assessment of patients with severe AS undergoing TAVI. Animal models have clearly demonstrated significant coronary physiological changes in severe AS [23], characterized by increased resting flow and increased microvascular resistance, which complicate functional lesion assessment. Clinically, adjusted ischemic cut-offs (FFR ≤ 0.83) specifically validated in AS patients improve diagnostic accuracy [24], overcoming known limitations of traditional thresholds. Furthermore, age-related changes influence coronary hemodynamics, suggesting that patient age should influence the timing of PCI relative to TAVI [25]. Taken together, these findings highlight the need to tailor CT-FFR assessment algorithms for AS patients, and future research should focus on integrating age-specific and AS-specific physiological parameters to optimize preoperative assessment and guide precise clinical decision-making.

## Conclusions

In patients with severe AS who are proposed to undergo TAVI, CT-FFR based on preoperative CTA performs well in screen for coronary stenosis, thus reducing unnecessary coronary angiography in this high-risk population.

## Electronic supplementary material

Below is the link to the electronic supplementary material.

Supplementary Material 1

## Data Availability

The authors confirm that the data supporting the findings of this study are available within the article.
