# Supplementary material for: Two birds with one stone: pre-TAVI coronary CT angiography combined with FFR helps screen for coronary stenosis
Source: BMC Med Imaging. 2025 May 26;25:192. doi: 10.1186/s12880-025-01704-2 (PMC12107836; doi:10.1186/s12880-025-01704-2)
Supplement: Supplementary file 1 — Supplementary Material 1 [file 12880_2025_1704_MOESM1_ESM.docx]

**Supplementary Table 1.** Additional diagnostic performance of cCTA and CT-FFR.

|  | Kappa value | LR+ | LR- | Youden index |
| --- | --- | --- | --- | --- |
| Patient cCTA | 0.247 | 1.878 | 0.662 | 0.244 |
| Patient CT-FFR | 0.508 | 2.549 | 0.206 | 0.525 |
| Vessels cCTA | 0.226 | 2.152 | 0.545 | 0.326 |
| Vessels CT-FFR | 0.422 | 3.359 | 0.291 | 0.545 |
| LM cCTA | 0.085 | 3.497 | 0.583 | 0.357 |
| LM CT-FFR | 0.263 | 4.124 | 0.209 | 0.631 |
| LAD cCTA | 0.003 | 1.007 | 0.991 | 0.004 |
| LAD CT-FFR | 0.173 | 1.476 | 0.643 | 0.204 |
| LCX cCTA | 0.455 | 4.550 | 0.278 | 0.600 |
| LCX CT-FFR | 0.521 | 4.876 | 0.212 | 0.655 |
| RCA cCTA | 0.113 | 1.805 | 0.642 | 0.248 |
| RCA CT-FFR | 0.558 | 4.200 | 0.200 | 0.640 |

*cCTA*, coronary computed tomography angiography; *CT-FFR*, computed tomography-fractional flow reserve; *LAD*, left anterior descending; *LCX*, left circumflex; *LM,* left main coronary artery; *LR+*, positive likelihood ratio; *LR-*, negative likelihood ratio; *RCA,* right coronary artery.
